# FOLFOXIRI plus cetuximab as conversion therapy for unresectable RAS/BRAF wild-type left-sided colorectal cancer with liver-limited metastases: a prospective dual-center pilot study

**DOI:** 10.3389/fonc.2024.1375906

**Published:** 2024-04-04

**Authors:** Wenwei Yang, Dong Chen, Yaru Niu, Guifu Wu, Zhangkan Huang, Xinyu Bi, Hong Zhao, Xu Che, Yongkun Sun

**Affiliations:** ^1^ Department of Medical Oncology, National Cancer Center/National Clinical Research Center for Cancer/Cancer Hospital, Chinese Academy of Medical Sciences and Peking Union Medical College, Beijing, China; ^2^ Department of Hepatobiliary and Pancreatic Surgery, National Cancer Center/National Clinical Research Center for Cancer/Cancer Hospital and Shenzhen Hospital, Chinese Academy of Medical Sciences and Peking Union Medical College, Shenzhen, China; ^3^ Department of Medical Oncology, Beijing Chaoyang District Sanhuan Cancer Hospital, Beijing, China; ^4^ Department of Hepatobiliary Surgery, National Cancer Center/National Clinical Research Center for Cancer/Cancer Hospital, Chinese Academy of Medical Sciences and Peking Union Medical College, Beijing, China; ^5^ Department of Pancreatic and Gastric Surgery, National Cancer Center/National Clinical Research Center for Cancer/Cancer Hospital, Chinese Academy of Medical Sciences and Peking Union Medical College, Beijing, China

**Keywords:** metastatic colorectal cancer, FOLFOXIRI, cetuximab, liver metastases, conversion therapy

## Abstract

**Purpose:**

To explore the efficacy and safety of FOLFOXIRI plus cetuximab regimen as conversion therapy for patients with unresectable RAS/BRAF wild-type colorectal liver-limited metastases (CLM).

**Patients and methods:**

This was a dual-center, phase II trial with the rate of no evidence of disease (NED) achieved as the primary endpoint. All enrolled patients with initially unresectable left-sided RAS/BRAF wild-type colorectal liver-limited metastases received a modified FOLFOXIRI plus cetuximab regimen as conversion therapy.

**Results:**

Between October 2019 and October 2021, fifteen patients were enrolled. Nine patients (60%) achieved NED. The overall response rate (ORR) was 92.9%, and the disease control rate (DCR) was 100%. The median relapse‐free survival (RFS) was 9 (95% CI: 0–20.7) months. The median progression-free survival (PFS) was 13.0 months (95% CI: 5.7-20.5), and the median overall survival (OS) was not reached. The most frequently occurring grade 3-4 adverse events were neutropenia (20%), peripheral neurotoxicity (13.3%), diarrhea (6.7%), and rash acneiform (6.7%).

**Conclusion:**

The FOLFOXIRI plus cetuximab regimen displayed tolerable toxicity and promising anti-tumor activity in terms of the rate of NED achieved and response rate in patients with initially unresectable left-sided RAS/BRAF wild-type CLM. This regimen merits further investigation.

## 1 Introduction

Colorectal cancer (CRC) remains a major health problem, ranking second in cancer-related deaths worldwide (1). Approximately 25% of CRC patients present with liver metastases at the time of diagnosis, and half of patients who received radical resection of CRC might subsequently develop recurrence in the liver (2). Notably, patients with colorectal liver-limited metastases (CLM) are considered a specific subgroup of metastatic colorectal cancer (mCRC), as they could benefit from a curative strategy, which offers a superior 5-year survival rate than palliative chemotherapy (3–6).

The management of CLM patients is a great challenge for oncologists, as about 80% of them are initially unresectable because of the number, size, or location of liver metastases (3). However, because of the optimal integration of systemic and locoregional treatments, including surgery, thermal ablation, stereotactic ablative body radiotherapy, and embolization techniques, an increasing number of patients with CLM achieve tumor downstaging and complete tumor removal with no evidence of disease (NED) after conversion therapy.

Recently, guidelines recommended an intensified regimen as the preferable option for patients with initially unresectable or borderline resectable CLM to induce earlier and deeper tumor shrinkage (7, 8). The standard conversion therapy regimens for CLM include doublet regimens plus an anti-EGFR monoclonal antibody for left-sided RAS wild-type patients and the triplet FOLFOXIRI (fluorouracil, leucovorin, oxaliplatin, and irinotecan) plus bevacizumab for right-sided or RAS/BRAF-mutated subgroup (9–12). Several clinical trials have demonstrated that the triplet chemotherapeutic regimen, consisting of fluorouracil (5-FU)/leucovorin, irinotecan, and oxaliplatin, could bring higher response rate and resection rate for patients with mCRC compared to the doublet regimens (FOLFOX or FOLFIRI) (13–15). Besides, for left-sided RAS/BRAF wild-type mCRC, incorporating anti-EGFR agents (e.g., cetuximab) into chemotherapy regimens can enhance efficacy. Therefore, the combination of triplet chemotherapy with an anti-EGFR agent might be a feasible conversion therapy for patients with unresectable left-sided RAS/BRAF wild-type mCRC.

At the time of designing our study, it is generally acknowledged by most scholars that a higher response rate is associated with a higher conversion rate. However, due to the toxicity and uncertain benefit, only a small number of patients received intensified triplet chemotherapy with an anti-EGFR agent regimen in clinical practice. Several studies evaluated the efficacy and safety of triplet chemotherapy plus anti-EGFR antibodies as conversion therapy for mCRC patients ongoing at that time, such as the TRIPLETE and TRICE study (16, 17). There is no consensus on conversion therapy in RAS/BRAF wild-type left-sided mCRC patients.

To address this gap, we conducted a dual-center, pilot phase II study to evaluate the efficacy and safety of triplet chemotherapy plus cetuximab regimen as conversion therapy for initially unresectable left-sided RAS/BRAF wild-type mCRC patients with liver-limited metastases.

## 2 Patients and methods

### 2.1 Patients

This is a nonrandomized pilot study conducted at two centers in China, including Cancer Hospital Chinese Academy of Medical Sciences and Cancer Hospital & Shenzhen Hospital, Chinese Academy of Medical Sciences and Peking Union Medical College.

Main inclusion criteria were as follows: age between 18 and 65 years old; ECOG performance status score of 0 to 1; histologically confirmed adenocarcinoma of the left-sided colon or rectum; liver metastasis confirmed with imaging and/or histopathological examinations; liver metastasis was considered initially unresectable by multidisciplinary team (MDT), RAS and BRAF wild-type; no prior treatment for colorectal cancer and liver metastasis; imaging confirmed without other organ metastases besides liver; and adequate bone marrow, hepatic, and renal function. The main exclusion criteria were any extrahepatic metastatic disease and recurrence of primary tumors. The definition of unresectable CLM is the patients who could not achieve NED status through optimal integration of locoregional treatments, including surgery, thermal ablation, stereotactic ablative body radiotherapy, and embolization techniques, and the remnant liver volume under 30%.

The study was done in accordance with the Declaration of Helsinki, and the protocol was approved by the local ethics committees of participating sites. All patients provided their written informed consent before enrollment.

### 2.2 Treatments

Patients received cetuximab (500 mg/m^2^ on day 1) plus modified FOLFOXIRI (irinotecan 150 mg/m^2^, oxaliplatin 85 mg/m^2^, and a continuous infusion of 5-FU at a dose of 2400 mg/m^2^ over 46 hours). Treatment was repeated every two weeks until disease progression or unacceptable toxicity or resectability or up to a maximum of 12 cycles. Dose reductions were allowed for severe drug-associated toxicities (≥ grade 3 non-hematological or grade 4 hematological toxicity). The efficacy was evaluated every three cycles, and the resectability was also evaluated at the same time. Patients who had lesions that were radically resectable after evaluation will receive surgery. After radical resection of metastases, continuing chemotherapy as adjuvant treatment was recommended for a total of 12 perioperative cycles. However, according to the postoperative physical conditions of each patient, the maintenance regimen could be administered after surgery. For patients not achieving NED after 12 cycles of induction therapy, maintenance treatment with 5-FU and cetuximab was continued until disease progression.

### 2.3 Study endpoints

The primary endpoint was the rate of NED achieved. Secondary endpoints were objective response rate (ORR), disease control rate (DCR), early tumor shrinkage (ETS), depth of response (DpR), relapse‐free survival (RFS), progression-free survival (PFS), overall survival (OS), and safety.

The rate of NED achieved was defined as the proportion of patients achieving R0 resection, complete remission, or macroscopically complete ablation of all tumor lesions. ORR was defined as the proportion of patients with complete response (CR) and partial response (PR) according to RECIST version 1.1, and DCR was defined as the percentage of patients experiencing CR, PR, and stable disease (SD). ETS was defined as at least a 20% decrease in the sum of the longest diameters of the RECIST target lesions at first reassessment compared to baseline, while DpR was defined as the maximum percentage of tumor shrinkage based on the sum of the longest diameters of target lesions according to RECIST v1.1 at the lowest point compared to baseline values. RFS was defined as the period from NED achieved to recurrence. PFS was defined as the time from the date of the first administration of this regimen to the date of the first documented disease progression or death due to any cause. OS was defined as the time from the date of beginning receiving this regimen to the date of death resulting from any cause.

### 2.4 Efficacy and safety assessments

The initial imaging was conducted within 21 days before treatment started. Tumor response was assessed every three cycles by chest and abdominopelvic computed tomography (CT) or magnetic resonance imaging (MRI) of the liver according to Response Evaluation Criteria in Solid Tumors, version 1.1 (RECIST v1.1). Experienced radiologists carried out the response evaluation. The radiologists were not involved in the study’s conduct. The resectablity was evaluated by MDT.

The surgery encompasses radical resection of primary colorectal cancer with or without concurrent removal of metastatic tumors. Complete thermal ablation was allowed for liver metastases. Surgery or thermal ablation was performed with curative intent. According to the surgical margins, surgical resection was classified as complete resection (R0), microscopic residual tumor (R1), and macroscopic residual tumor (R2). R0 resection denotes the absence of cancer cells at the margin under microscopic examination. R1 resection signifies the removal of all visible lesions but detecting cancer cells at the margin under microscopic evaluation. R2 resection refers to visible tumor tissue remaining at the margin.

Adverse events (AEs) were graded based on the National Cancer Institute Common Terminology Criteria for Adverse Events version 4.0. Patients receiving at least one cycle of treatment underwent safety evaluation.

### 2.5 Gene mutation detection

Tumor tissue specimens from the primary tumor or metastases were used to detect KRAS, NRAS, and BRAF mutations by next-generation sequencing.

### 2.6 Statistical analysis

The study was designed as an exploratory, pilot study. Approximately 20 patients were planned for enrollment. The ORR, DCR, ETS, and DpR analysis was performed on all patients experiencing at least one reexamination. AEs were assessed in patients who received at least one cycle of treatment. The median follow-up period with the 95% CI was calculated by the reverse Kaplan-Meier method. Survival endpoints, PFS, and OS were analyzed by the Kaplan-Meier method, expressed as medians, and compared with the log-rank test and Cox regression (with hazard ratios [HRs] and 95% CIs indicated). Moreover, the Kaplan–Meier method was used to evaluate survival endpoints (i.e., PFS and OS) and establish the survival curves. The log-rank test assessed any significant differences in PFS stratified by whether achieving NED. A two-sided p-value < 0.05 was considered statistically significant. All statistical analyses were performed using software SPSS version 29.0 (IBM, Armonk, NY, USA).

## 3 Results

### 3.1 Patient characteristics

The cutoff date for the analysis was Oct 31, 2023. In all, 15 patients were enrolled between October 2019 to October 2021. The patients’ demographic and clinicopathological characteristics at baseline are summarized in **Table 1**. The median age was 58 (38–68), and most patients were male (80%). Four (26.7%) patients had an ECOG PS of 0, and 9 (60%) had an ECOG PS of 1. All mCRC patients presented with synchronous liver metastases and had a left-sided primary tumor. The primary tumor location was on the left-sided colon in 9 and on the rectum in 7 patients (one patient had two primary tumors). Ten patients had five or more liver metastases, and eight patients had a maximum size of liver metastasis ≥5cm. All patients were evaluated for RAS/BRAF mutation status and had RAS/BRAF wild-type tumors. The MMR status of 6 patients was pMMR, and the other nine patients were unknown. The median number of cycles of FOLFOXIRI plus cetuximab regimen administered was four (range, 1–10). Nine of 15 patients experienced primary resection in this study.

**Table 1:** Demographic and clinicopathological characteristics at baseline.

Characteristic	No. of patients	%
**Entire population**	15	100
**Median (range) age, years**	58(38-68)	
Sex		
Male	12	80
Famale	3	20
ECOG PS		
0	4	26.7
1	9	60
2	2	13.3
Primary tumor site		
Left colon	9	60
Rectum	7	46.7
Time to metastases		
Synchronous	15	100
Metachronous	0	0
No. of liver metastases		
>15	4	26.7
5-15	6	40
<5	5	33.3
Maximum size of liver metastases, cm		
≥5	8	53.3
<5	7	46.7
MMR status		
pMMR	6	40
dMMR	0	0
NA	9	60
RAS/BRAF status		
Wild type	15	100
Mutant type	0	0

### 3.2 Response to the treatment

Of the 15 enrolled patients, 14 were evaluated for response and resectability. One patient could not be evaluated for the response and was excluded from the efficacy analysis because he lacked assessment of tumor response after the third cycle due to COVID-19. The median number of cycles before surgery was 4.5 (range 1-10). PR was achieved in 13 patients, and one obtained an SD. The ORR was 92.9%, and the DCR was 100%.

**Figure 1:**
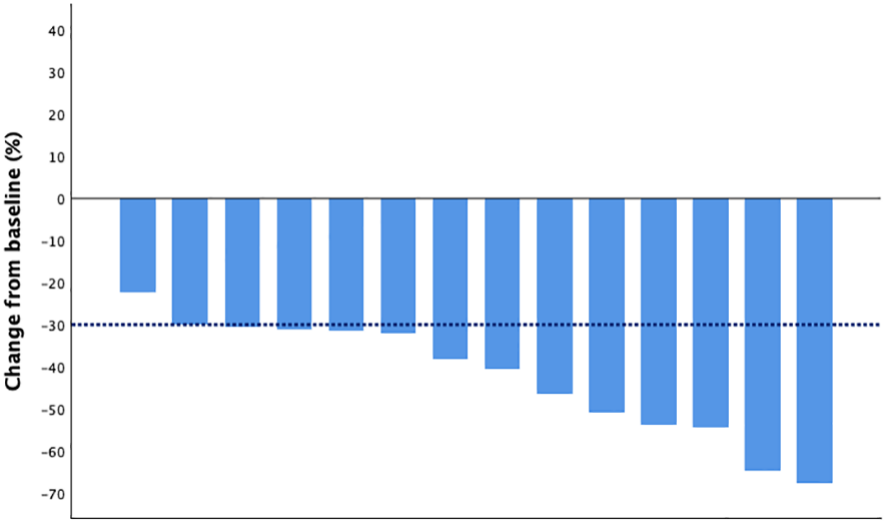
Depth of response. Each bar represents the best response for one patient during treatment.

The DpR and ETS were assessed in 14 patients. The median DpR was -39.3% (range from -67.6% to -22.2%), and 12 (80%) patients achieved ETS. The waterfall plot of the depth of response is shown in **Figure 1**, and the indices of efficacy outcomes are listed in **Table 2**.

**Table 2:** Efficacy outcomes.

Variable	No. of patients (n=15)	%
**ORR, n (%)**	13	92.9
CR	0	
PR	13	
SD	1	
PD	0	
NE	1	
**DCR, n (%)**	14	100
**PFS, median (95%CI), months**	13.0 (5.7-20.5)	
**OS, median (95%CI), months**	Not reached	
**NED achieved, n (%)**	9	60%
**ETS, n (%)**	12	80%
**DpR, median (range), %**	-39.3 (-67.6 to -22.2)	

ORR, objective response rate; CR, complete response; PR, partial response; SD, stable disease; PD, progressive disease; NE, not evaluable; DCR, disease control rate; PFS, progression-free survival; OS, overall survival; CI, confidence interval; NED, no evidence of disease; ETS, early tumor shrinkage; DpR, depth of response.

Only 14 patients were evaluable for short-term efficacy.

### 3.3 Conversion therapy

Out of 15 patients, nine (60%) achieved NED status. Seven patients achieved concurrent or staged resection of primary and metastatic lesions, and two experienced resection of primary tumor combined with thermal ablation of liver metastases.

After a median follow-up of 30 months, all these nine patients had recurrence. The median RFS was 9 (95% CI: 0–20.7) months. Eight out of 9 patients (88.9%) had disease recurrence in the liver, and only one had recurrence outside the liver (the rectal fascia). At the analysis date, eight out of the nine patients who achieved NED were alive.

### 3.4 PFS and OS

**Figure 2:**
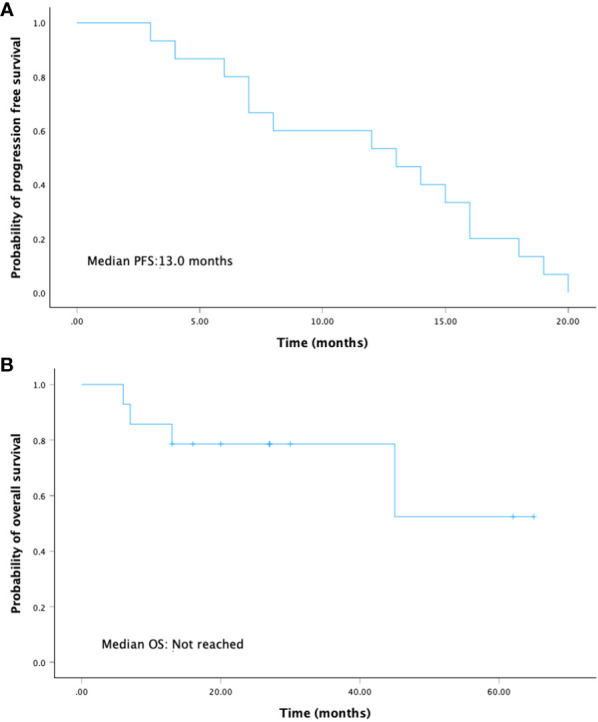
Kaplan-Meier curves for **(A)** progression-free survival and **(B)** overall survival.

With a median follow-up of 30 months (range 13-65), there were 10 out of 15 (66.7%) patients alive and 5 out of 15 (33.3%) patients who died because of the disease progression. The median PFS was 13.0 months (95% CI: 5.7-20.5), and the median OS was not reached (**Figure 2**).

**Figure 3:**
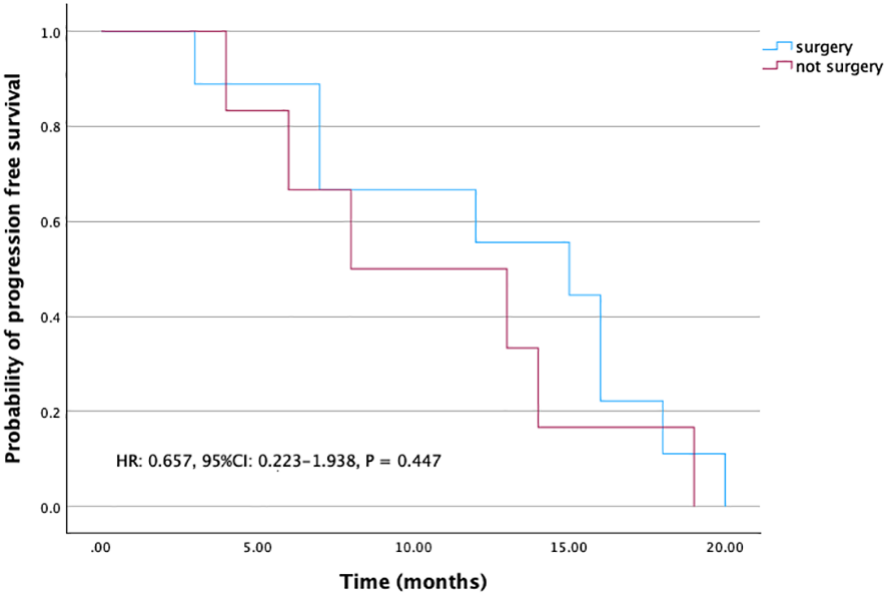
Kaplan-Meier curves for PFS in patients with surgery or without surgery.

For those patients who achieved NED, PFS was 15 months (95% CI: 6.2-23.8), whereas PFS was eight months (95% CI: 0-16.4) for those patients who were not resected (**Figure 3**). Though this difference is not significant, patients who achieved NED tended to experience longer PFS.

### 3.5 Treatment toxicity

The treatment-relevant adverse events (AEs) reported during the treatment period are presented in **Table 3**. All 15 patients were evaluated for safety. Neutropenia (80%), fatigue (73.3%), and rash acneiform (73.3%) were the most common AEs observed in these patients, followed by nausea (60%) and peripheral neurotoxicity (60%). The most frequently occurring grade 3 or 4 AEs were neutropenia (3 patients, 20%), peripheral neurotoxicity (2 patients, 13.3%), diarrhea (1 patient, 6.7%), and rash acneiform (1 patient, 6.7%). After dose reduction, there was a significant reduction in the proportion of patients who experienced severe AEs. There was no treatment-related death in all patients.

**Table 3:** Most common adverse events (maximum grade per patient per event).

Adverse event	Any grade, n(%)	Grade 3 or 4, n(%)
Haematologic toxicity		
Anemia	4 (26.7)	0
Thrombocytopenia	6 (40)	0
Neutropenia	12 (80)	3 (20)
Febrile neutropenia	0	0
Non-haematologic toxicity		
Nausea	9 (60)	0
Vomiting	5 (33.3)	0
Diarrhea	6 (40)	1 (6.7)
Constipation	0	0
Peripheral neurotoxicity	9 (60)	2 (13.3)
Fatigue	11 (73.3)	0
Alopecia	6 (40)	0
Hand-foot syndrome	3 (20)	0
Rash acneiform	11 (73.3)	1 (6.7)
Stomatitis	1 (6.7)	0
Hypomagnesemia	2 (13.3)	0
Hypocalcemia	0	0
Hypokalemia	0	0
ALT/AST increased	7 (46.7)	0

## 4 Discussion

The liver is the most frequently involved organ in mCRC (18). Patients with liver-limited metastases from colorectal cancer represent a particular subgroup in which patients with surgically resectable metastatic lesions can be treated with potentially curative multidisciplinary strategies and achieve higher 5-year survival rates of 28%–39% than palliative chemotherapy (3–5). The 10-year survival rate of these patients with hepatic metastases surgically removed is about 17% (19).

However, not all CRC patients with liver metastases are candidates for surgical resection initially due to functional hepatic reserve after resection, tumor location, performance status, and comorbidities. Approximately 80% of these patients are considered initially unresectable (3). In recent years, evidence has grown that preoperative chemotherapy can downsize tumors and facilitate subsequent radical resection (3, 20–22). Because of the effective systematic therapy and local treatment (e.g., surgery, thermal ablation, stereotactic ablative body radiotherapy, embolization techniques), an increasing number of CRC patients with hepatic metastases after conversion therapy have the possibility of completely removing tumors and achieving the NED (23, 24). Patients who experience NED status will have prolonged survival time.

There is an urgent need for effective conversion therapy regimens for CRC patients with liver metastases. Chemotherapeutic doublet regimens combined with anti-VEGF or anti-EGFR monoclonal antibodies represent the standard of care in untreated mCRC (25–29). As an anti-EGFR monoclonal antibody, cetuximab blocks the binding of epidermal growth factor and other ligands, inhibiting the cellular pathways involved in cell proliferation, angiogenesis, and metastasis (30). Mutations in RAS, BRAF, and PI3K, the critical signaling effectors downstream of EGFR, are associated with resistance to cetuximab (31). Multiple retrospective studies have confirmed that patients harboring RAS and BRAF mutations could not benefit from anti-EGFR therapies. The FIRE-4.5 trial is the first prospective study to verify that FOLFOXIRI plus cetuximab did not induce a higher ORR in first-line treatment of BRAF V600E-mutant mCRC when compared with FOLFOXIRI plus bevacizumab (32). Evidence suggests that combining cetuximab with doublet chemotherapy brings survival benefits for patients with RAS/BRAF wild-type mCRC (33, 34). To further intensify efficacy, a triplet regimen, FOLFOXIRI, including 5-FU, irinotecan, and oxaliplatin, was developed and yielded higher response and resection rates (2, 13). Therefore, for RAS/BRAF wild-type mCRC patients, several trials explored the FOLFOXIRI plus anti-EGFR antibody (cetuximab or panitumumab) regimen as conversion therapy and preliminarily showed its promising efficacy (35, 36).

Several studies have been conducted on anti-EGFR monoclonal antibody plus triplet chemotherapy regimen as first-line or conversion therapy for RAS/BRAF wild-type mCRC patients. In these studies, the ORR ranged from 66.7% to 95.5% (36, 37). The median PFS ranged from 9.3 months to 16.0 months (38, 39), and the median OS ranged from 24.7 months to 55 months (40, 41). In our study, the ORR was 92.9%, and the median PFS was 13.0 months, conformed to the previous studies. A triplet regimen combined with anti-EGFR monoclonal antibody is more applicable for conversion therapy in mCRC patients.

However, whether the FOLFOXIRI plus anti-EGFR antibody regimen can be used as an upfront treatment for RAS/BRAF wild-type mCRC patients remains controversial due to negative results from several clinical trials. TRIPLETE study, a prospective phase III trial, investigated the efficacy of FOLFOXIRI plus panitumumab regimen compared to FOLFOX plus panitumumab regimen in untreated patients with unresectable RAS/BRAF wild type mCRC. However, FOLFOXIRI plus panitumumab regimen did not bring additional survival benefits compared to doublet chemotherapy plus panitumumab (42). Another phase II randomized controlled trial, the TRICE study (NCT03493048), compared the efficacy and safety of cetuximab plus FOLFOXIRI regimen versus cetuximab plus FOLFOX regimen in the first-line treatment of patients with RAS wild-type initially unresectable colorectal cancer liver metastasis. From January 2018 to December 2022, 146 patients were recruited. Updated results were presented at the 2023 ESMO Congress (17). It is reported that though intensified systemic chemotherapy in RAS wild-type metastatic CRC patients offered a better DpR, there was no significant difference in ORR, PFS, and R0 resection rate with a higher incidence of grade 3-4 neutropenia and diarrhea. The unselected patient population may have contributed to the negative results. Several clinical trials are ongoing to continue exploring this regimen’s clinical value.

Conversion therapy is necessary for mCRC patients, especially for patients with CLM. CLM is heterogeneous to other mCRC, with distinct biological behaviors (43). Several studies have confirmed that the R0 resection of CLM can significantly prolong the survival time, highlighting the importance of designing optimal conversion therapy for CLM patients (44). According to a meta-analysis, the pooled R0 resection rate in CLM patients was 60%. Among all included studies, five were conducted to evaluate the R0 resection rate of CLM after triplet chemotherapy plus an anti-EGFR antibody regimen, and the R0 resection rate ranged from 60% to 84% (45). In our study, the rate of NED achieved is 60%. Patients who achieved NED had a numerically longer PFS compared with those who did not achieve NED, and their median OS did not reach. Other indices for conversion therapy, such as DpR and ETS, also reflect the rate and magnitude of tumor downsizing. We gained promising DpR and ETS from our study, which demonstrated that triplet chemotherapy plus cetuximab might become a preferable choice as conversion therapy for CLM patients.

Our final goal is to prolong overall survival, and only by converting tumor shrinkage to R0 resection can patients experience longer survival time. Besides designing optimal treatment, it is also necessary to identify the patients who may benefit most from this regimen. Considering the favorable survival outcomes in patients with left-sided mCRC tumors receiving anti-EGFR agents, it could be reasonably inferred that RAS/BRAF wild-type left-sided CLM patients may benefit most from triplet chemotherapy plus anti-EGFR agent regimen (46). The DEEPER trial also indicated that compared to mFOLFOXIRI plus bevacizumab regimen, mFOLFOXIRI plus cetuximab regimen could be an ideal option for first-line chemotherapy with higher DpR and longer PFS in left-sided mCRC patients with RAS/BRAF wild-type (47). However, in the 2023 ESMO congress, the CAIRO5 study indicated that OS was not different between adding panitumumab versus bevacizumab to FOLFOX/FOLFIRI for initially unresectable left-sided RAS/BRAF V600E wild-type CRLM. Compared with chemotherapy plus bevacizumab, there was neither no improvement in PFS (10.8 months versus 10.4 months; p = 0.46)) or R0/R1 resection and/or ablation rate (58% versus 58%; p = 1.0) from chemotherapy plus anti-EGFR therapy in this population, but was associated with more toxicity (48). Further research needs to be conducted to identify the patients who may benefit most from this regimen.

Furthermore, we believe a strong relationship exists between the number of liver metastases and prognosis. In our study, 4 (26.7%) patients with liver metastases above 15, 6 patients (40%) with liver metastases between 5 and 15, 5 patients (33.3%) with liver metastases under 5. Neither of the patients with liver metastases above 15 achieved NED status. Four of the 6 (66.7%) patients with liver metastases between 5 and 15 achieved NED status. All patients (100%) with liver metastases under 5 achieved NED status. These data preliminarily verified this opinion. We also hold the opinion that liver metastases could be divided into two groups: huge mass type liver metastases with fewer numbers and multiple distributed metastases in both lobes of the liver. Patients with huge mass type liver metastases might be suitable for this intensified regimen with a better prognosis. Further investigations on screening appropriate patients for benefitting from this regimen s are needed.

Concerning safety, even though the dose of FOLFOXIRI chemotherapy was reduced to decrease toxicity, the triplet was still associated with increased toxicity compared with doublet regimens. In our study, the most common AEs were neutropenia (80%), fatigue (73.3%), and rash acneiform (73.3%). Neutropenia and peripheral neurotoxicity were the major grade ≥ 3 AEs reported in 3 and 2 patients, respectively. Grade ≥ 3 diarrhea and rash acneiform were both reported in 1 patient (6.7%), respectively. A similar incidence of AEs was documented in previous studies. The rate of grade 3 or 4 neutropenia ranged from 0 to 48.6% (37, 49). Grade≥ 3 diarrhea incidence ranged from 7.5% to 53.3% (36, 50). The reported incidence of grade 3/4 skin toxicity in patients with FOLFOXIRI plus anti-EGFR agent ranges from 0 to 33.3% (36, 37, 40). However, these AEs were generally manageable based on dose reduction. Overall, these studies indicated that, for triplet chemotherapy plus anti-EGFR antibodies regimen, appropriate management and supportive treatment are required for good tolerability.

Several limitations of our study should be considered. Firstly, the sample size of this study was relatively small. Secondly, this is a non-comparative study and only demonstrated the efficacy of FOLFOXIRI plus cetuximab regimen compared with historical controls rather than the control arm. A randomized controlled trial is needed to determine whether this regimen could bring patients survival benefits.

The best regimen for left-sided RAS/BRAF wild-type CLM remains a question. Some scholars suggest that the efficacy of doublet chemotherapy plus anti-EGFR agent regimen is enough for RAS/BRAF wild-type mCRC patients in first-line treatment, which enables them to have more chemotherapy regimen options in second or later-line treatments. Nevertheless, triplet chemotherapy plus anti-EGFR antibody is not without its applicable population. This regimen could be used chiefly for conversion therapy, and the target population is refined to younger patients with overt symptoms and a high tumor load or metastatic burden. In clinical practice, an appropriate individualized chemotherapy regimen should be designed based on both patient and tumor characteristics. The efficacy and safety of cetuximab combined with FOLFOXIRI for CLM are under active investigation. We look forward to these studies providing further insights.

## 5 Conclusion

In conclusion, the FOLFOXIRI plus cetuximab regimen offered a promising rate of NED achieved and response rate to initially unresectable left-sided RAS/BRAF wild-type CLM patients with tolerable toxicity, which might become an option for patients with initially unresectable CLM. This approach remains investigational at this stage, and its potential needs to be further studied.

## Data availability statement

The original contributions presented in the study are included in the article/**Supplementary Material**. Further inquiries can be directed to the corresponding authors.

## Ethics statement

The studies involving humans were approved by Ethics Committee of National Cancer Center/National Clinical Research Center for Cancer/Cancer Hospital, Chinese Academy of Medical Sciences and Peking Union Medical College. The studies were conducted in accordance with the local legislation and institutional requirements. The participants provided their written informed consent to participate in this study.

## Author contributions

WY: Data curation, Investigation, Software, Writing – original draft, Writing – review & editing. DC: Data curation, Methodology, Validation, Writing – original draft, Writing – review & editing. YN: Data curation, Validation. GW: Writing – review & editing. ZH: Writing – review & editing. XB: Writing – review & editing. HZ: Writing – review & editing. XC: Conceptualization, Funding acquisition, Project administration, Supervision, Writing – review & editing. YS: Conceptualization, Funding acquisition, Project administration, Supervision, Validation, Writing – review & editing.
