# Supplementary material for: FOLFOXIRI plus cetuximab as conversion therapy for unresectable RAS/BRAF wild-type left-sided colorectal cancer with liver-limited metastases: a prospective dual-center pilot study
Source: Front Oncol. 2024 Apr 4;14:1375906. doi: 10.3389/fonc.2024.1375906 (PMC11024419; doi:10.3389/fonc.2024.1375906)
Supplement: Supplementary file 2 [file DataSheet_2.doc]

PFS
Kaplan-Meier


Case Processing Summary	
group	Total N	N of Events	Censored	
			N	Percent	
1.00	15	15	0	0.0%	
Overall	15	15	0	0.0%	


Survival Table	
group	Time	Status	Cumulative Proportion Surviving at the Time	N of Cumulative Events	N of Remaining Cases	
			Estimate	Std. Error			
1.00	1	3.000	1.00	.933	.064	1	14	
	2	4.000	1.00	.867	.088	2	13	
	3	6.000	1.00	.800	.103	3	12	
	4	7.000	1.00	.	.	4	11	
	5	7.000	1.00	.667	.122	5	10	
	6	8.000	1.00	.600	.126	6	9	
	7	12.000	1.00	.533	.129	7	8	
	8	13.000	1.00	.467	.129	8	7	
	9	14.000	1.00	.400	.126	9	6	
	10	15.000	1.00	.333	.122	10	5	
	11	16.000	1.00	.	.	11	4	
	12	16.000	1.00	.200	.103	12	3	
	13	18.000	1.00	.133	.088	13	2	
	14	19.000	1.00	.067	.064	14	1	
	15	20.000	1.00	.000	.000	15	0	


Means and Medians for Survival Time	
group	Meana	Median	
	Estimate	Std. Error	95% Confidence Interval	Estimate	Std. Error	95% Confidence Interval	
			Lower Bound	Upper Bound			Lower Bound	Upper Bound	
1.00	11.867	1.450	9.024	14.709	13.000	3.864	5.426	20.574	
Overall	11.867	1.450	9.024	14.709	13.000	3.864	5.426	20.574	

a. Estimation is limited to the largest survival time if it is censored.	


OS
Kaplan-Meier


Case Processing Summary	
group	Total N	N of Events	Censored	
			N	Percent	
1.00	14	4	10	71.4%	
Overall	14	4	10	71.4%	


Survival Table	
group	Time	Status	Cumulative Proportion Surviving at the Time	N of Cumulative Events	N of Remaining Cases	
			Estimate	Std. Error			
1.00	1	6.000	1.00	.929	.069	1	13	
	2	7.000	1.00	.857	.094	2	12	
	3	13.000	1.00	.786	.110	3	11	
	4	13.000	.00	.	.	3	10	
	5	16.000	.00	.	.	3	9	
	6	20.000	.00	.	.	3	8	
	7	27.000	.00	.	.	3	7	
	8	27.000	.00	.	.	3	6	
	9	27.000	.00	.	.	3	5	
	10	27.000	.00	.	.	3	4	
	11	30.000	.00	.	.	3	3	
	12	45.000	1.00	.524	.226	4	2	
	13	62.000	.00	.	.	4	1	
	14	65.000	.00	.	.	4	0	


Means and Medians for Survival Time	
group	Meana	Median	
	Estimate	Std. Error	95% Confidence Interval	Estimate	Std. Error	95% Confidence Interval	
			Lower Bound	Upper Bound			Lower Bound	Upper Bound	
1.00	47.690	6.936	34.096	61.284	.	.	.	.	
Overall	47.690	6.936	34.096	61.284	.	.	.	.	

a. Estimation is limited to the largest survival time if it is censored.	
